# Retraction: Interleukin-1β-Induced Autophagy-Related Gene 5 Regulates Proliferation of Embryonic Stem Cell-Derived Odontoblastic Cells

**DOI:** 10.1371/journal.pone.0224332

**Published:** 2019-10-18

**Authors:** The PLOS ONE Editors

The corresponding author requested retraction of this article [1] due to concerns about the integrity of the data and the validity of the conclusions. The underlying data supporting the results in this article are not available.

The following specific concerns were noted about the reported results:

- The brightness and contrast on the western blot figures in the article do not allow a confirmation of the integrity of the results as presented.
- The β-tubulin blots appear similar in Fig 7 panels A & D, panels B & E, and panels C & F.
- BrdU and apoptosis microscopy images in the following panels of Figs 4, 5, and 6 appear to report the same data:
  - BrdU and apoptosis images in Fig 4B, Fig 5B, and 6B for experiments i, ii, iii
  - BrdU images in panel vi of Fig 4B and 6B, which represent different experiments
  - Apoptosis images in panel vi of Fig 4B, Fig 5B, and Fig 6B, which represent different experiments

The authors were unable to provide any clarifications about these issues.

Aichi Gakuin University is investigating this work. The investigating committee has tentatively confirmed that there are concerns about the quality of the western blot images reported in this article and that the underlying data are no longer available.

In light of the above concerns, the *PLOS ONE* Editors retract this article.

NH, HY, RK, AK, TM, KN, and MM agree with the retraction. NO and TH did not respond or could not be reached.
